# *Torulaspora delbrueckii* May Help Manage Total and Volatile Acidity of Santorini-Assyrtiko Wine in View of Global Warming

**DOI:** 10.3390/foods12010191

**Published:** 2023-01-01

**Authors:** Georgios Sgouros, Athanasios Mallouchos, Dimitra Dourou, Georgios Banilas, Ioanna Chalvantzi, Yiannis Kourkoutas, Aspasia Nisiotou

**Affiliations:** 1 Institute of Technology of Agricultural Products, Hellenic Agricultural Organization “Dimitra”, 14123 Lykovryssi, Greece; 2 Laboratory of Applied Microbiology and Biotechnology, Department of Molecular Biology and Genetics, Democritus University of Thrace, 68100 Alexandroupolis, Greece; 3 Laboratory of Food Chemistry and Analysis, Department of Food Science and Human Nutrition, Agricultural University of Athens, 11855 Athens, Greece; 4 Department of Wine, Vine and Beverage Sciences, University of West Attica, 12243 Athens, Greece

**Keywords:** non-*Saccharomyces*, wine fermentation, yeast starter cultures, *Torulaspora delbrueckii*, *Lachancea thermotolerans*, wine chemical profile, volatiles

## Abstract

Non-*Saccharomyces* (NS) yeasts are gaining popularity in modern winemaking for improving wine quality. Climate change is one of the biggest challenges winegrowing now faces in warm regions. Here, *Lachancea thermotolerans* LtS1 and *Torulaspora delbrueckii* TdS6 combined with *Saccharomyces cerevisiae* ScS13 isolated from Assyrtiko grapes from Santorini island were evaluated in grape must fermentation with the aim to mitigate major consequences of temperature rise. Different inoculation protocols were evaluated, including simultaneous and sequential mixed-strain inoculations, displaying significant variation in the chemical and kinetic characteristics. Both LtS1 and TdS6 could raise the titratable acidity (TA). TdS6 also reduced the volatile acidity (VA) and was thus chosen for further evaluation in microvinifications and pilot-scale fermentations. Consistent with lab-scale trials, sequential inoculation exhibited the longest persistence of TdS6 resulting in minimum VA levels. Diethyl succinate, ethyl propanoate, and ethyl isobutyrate were significantly increased in sequential inoculations, although a decline in the net total ester content was observed. On the other hand, significantly higher levels of TA, succinic acid, and 2-methylpropanoic were associated with sequential inoculation. The overall performance of TdS6 coupled with a high compatibility with *S. cerevisiae* suggests its use in the fermentation of Santorini-Assyrtiko or other high sugar musts for the production of structured dry or sweet wines.

## 1. Introduction

The yeasts residing on grape berries are of high importance in winemaking. The majority of these vine-associated populations are brought into the winery along with the harvested grapes, while winery yeasts may find their way back to the vineyard [1]. This dynamic yeast consortium of the grape-wine biological system contributes to wine quality, and has been recently recognized as a potential contributor to the terroir expression of wines. Bokulich et al. [2] by applying metagenomics targeting the whole fungal community were among the first to demonstrate that geographical influence might drive regional grape-associated microbial biodiversity. Recently, Chalvantzi et al. [3] focusing exclusively on the wine yeast populations, showed that different viticultural appellations may indeed possess distinct species diversity characterized by particular enological features. These results endorse the plausible effect of indigenous yeasts on the regional typicity of wines, the so-called microbial terroir effect. However, the addition of commercial *Saccharomyces cerevisiae* strains at high concentrations into must immediately after grape crushing overwhelms the indigenous yeast populations (both *S. cerevisiae* and non-*Saccharomyces* species) and dominates the fermentation course. Despite certain advantages, the use of yeast starters may thus deprive wines of the microbial *terroir* influence that spontaneous fermentation provides, often diminishing the organoleptic and chemo-sensory spatial variability of wines [4]. Non-*Saccharomyces* (NS) species, in particular, play a major role in the formation of critical metabolites that influence wine taste and may shape the regional character of wines [5].

The grape-associated yeast microbiota contains a diverse range of species and strains, mainly of the genera *Hanseniaspora*, *Starmerella*, *Lachancea*, *Torulaspora*, and *Metschnikowia* [3,5]. As more data become available, it appears that several NS yeast species may offer intriguing enological characteristics to be used along with *S. cerevisiae* as starters in winemaking [6]. Significant research has been conducted recently, as reviewed by Lappa et al. [7], to improve enological procedures and enhance the varietal character utilizing multi-starter fermentation processes including NS yeasts.

Assyrtiko is a white grape variety originating in Santorini, a small volcanic island in the Cyclades, in the south Aegean Sea, of millennial tradition in viticulture and winemaking. Assyrtiko has been cultivated in Santorini since ancient times, while currently it has been widely distributed throughout Greece and also in some other countries such as Italy, Australia and the USA. While many Assyrtiko wines from various regions are becoming more popular, the terroir-driven Assyrtiko-Santorini volcanic wines are distinctive in that they are structured on high levels of crisp acidity, savorous density and enthralling minerality. However, this unique terroir is at risk of significant degradation due to climate change. Global warming in particular is expected to have detrimental effects in this iconic wine appellation [8], as in other viticultural regions of warm climate worldwide. The rise in temperature is expected to lower the acidity and increase the sugar content of berries, resulting in unbalanced wines with problems in shelf stability and also with higher alcohol content prone to increased volatile acidity [9]. Thus, means for acidification and lowering volatile acidity may become unavoidable. To counteract these issues, tartaric acid is frequently added. However, the outcomes are inconsistent and often involve problems of unbalanced wines [10]. Utilizing selected yeasts that may produce organic acids to boost wine acidity, the so-called biological acidification, and concomitantly to produce minimal volatile acidity during the alcoholic fermentation may serve as an appealing and affordable alternative [11,12].

The aim of this study was to evaluate the performance of selected indigenous yeasts of the species *Lachancea thermotolerans*, *Torulaspora delbrueckii* and *S. cerevisiae* in various mixed-culture fermentation schemes towards biological acidification and benefiting the volcanic Assyrtiko-Santorini terroir. Biological acidification of wine during fermentation with the use of *L. thermotolerans* has been reported previously [11,13,14]. *T. delbrueckii* is probably the most widely used non-*Saccharomyces* yeast species in winemaking due to its ability to improve wine quality through enhancing aroma profile or lowering acetaldehyde content [15]. The species is characterized by a great genetic/phenotypic genotypic variability that may partially explain how various strains may differently affect wine quality parameters [15,16]. Here, we show that a *T. delbrueckii* Santorini vineyard isolate (strain TdS6) may be applied in winemaking to increase the titratable acidity of wines and at the same time to substantially reduce the volatile acidity. This is of particular importance in the case of Assyrtiko-Santorini and potentially of other wines of warm regions in view of global warming’s expected detrimental effects on wine-growing.

## 2. Materials and Methods

### 2.1. Yeast Strains

The yeast strains *L. thermotolerans* LtS1, *T. delbrueckii* TdS6, and *S. cerevisiae* ScS13 were recovered from naturally fermenting Assyrtiko grape must from vineyards of the Santorini island in the Aegean Sea, Greece. Strains were previously selected based on positive enological characteristics such as ethanol and SO_2_ resistance, acetic acid production, H_2_S production and fermentation power (unpublished data). A commercial *S. cerevisiae* starter (Lalvin QA23, Lallemand Inc., Montreal, QC, Canada) was also used as reference. Restriction enzyme analysis of the 5.8S ITS rDNA region was applied for species level identification as described elsewhere [17]. *S. cerevisiae* isolates were genotyped by interdelta region analysis with the primer pair delta 12-delta 21 [18]. Genotyping of *L. thermotolerans* was accomplished by the tandem repeat-tRNA method using the primer pair TtRNASc/ISSR-MB [19]. For the discrimination of *T. delbrueckii* isolates, RAPD analysis with the R5 primer was used [20].

### 2.2. Small-Scale Laboratory Fermentations in Sterile Must

Fermentations were performed in triplicate at 20 °C under static conditions in 1000 mL flasks containing 750 mL pasteurized (70 °C, 10 min) Assyrtiko grape must from Santorini (sugars 229 g/L; pH 3.08; titratable acidity 8.5 g/L, as tartaric acid; yeast assimilable nitrogen 240 mg/L). Must was supplemented with 30 ppm SO_2_ as potassium metabisulfite. Flasks were sealed with fermentation locks filled with glycerol. Yeast inocula were grown in must (26 °C, 18 h, 225 rpm) and added at a concentration of 6 Log CFU/mL according to the following inoculation protocols: single inoculation of the indigenous *S. cerevisiae* ScS13 strain (hereafter referred to as IS), simultaneous inoculation (SM) of ScS13 with *L. thermotolerans* LtS1 (SML) or *T. delbrueckii* TdS6 (SMT), sequential inoculation (SQ) of TdS6 (SQT) or LtS1 (SQL) followed by *S. cerevisiae* ScS13 at 1% vol. ethanol, and single inoculation of the commercial *S. cerevisiae* strain (CS). The fermentation course was monitored by measuring weight loss.

### 2.3. Microvinifications

Fermentations were carried out at 20 °C in triplicate in 10 L flasks filled with 6 L of Assyrtiko grape must from Santorini (sugars 223 g/L; pH 3.14; titratable acidity 8.0 g/L, as tartaric acid; yeast assimilable nitrogen 240 mg/L; 30 ppm SO_2_). Flasks were closed with airlocks containing glycerol. Yeast inocula were grown in must (26 °C, 18 h, 225 rpm) and added at a concentration of 6 Log CFU/mL into the IS, SMT, SQT and CS fermentations, as defined above. Duplicate spontaneous fermentations (SP) were also performed as reference. Density measurements were taken throughout the fermentation process.

### 2.4. Pilot-Scale Fermentations

Pilot scale fermentations were performed in duplicate (triplicate in IS) in 220-L tanks filled with 170 L of Assyrtiko-Santorini must (sugars 221 g/L; pH 3.13; titratable acidity 6.35 g/L, as tartaric acid; 50 ppm total SO_2_; initial yeast assimilable nitrogen 100.8 mg/L) at 18 °C. Forty g/hL of organic nitrogen was added into the grape must prior to yeast inoculation and another 40 g/hL of nitrogen in both organic and inorganic form were added at 50 g/L sugar depletion. Yeasts were grown on yeast extract peptone dextrose (YPD) agar at 26 °C and then resuspended in 1/4 strength Ringer’s solution. Yeasts were added at 6 Log CFU/mL in fermenting musts of the IS, SMT and SQT inoculation protocols, as above. Duplicate spontaneous fermentations (SP) were also performed for comparison reasons. Fermentation courses were monitored by density measurements.

### 2.5. Microbiological Analysis

Total yeasts, *S. cerevisiae*, and non-*Saccharomyces* kinetics were daily followed by plating on Wallerstein laboratory nutrient agar (WL), ethanol sulfite agar (ESA) and yeast extract–peptone–dextrose agar (YPD), and lysine medium agar (LA), respectively. Colonies were enumerated after incubation of plates at 28 °C (37 °C for YPD plates) for 2–5 days. *T. delbrueckii* and *S. cerevisiae* colonies recovered from the initial, middle and final stages of non-sterile fermentations were genotyped as described above. 

### 2.6. Chemical Analysis

Reducing sugars, total acidity, volatile acidity, pH, total SO_2_ and free SO_2_ were estimated as described in the Compendium of International Methods of Analysis of Musts and Wines [21]. The formol method [22] was applied for the estimation of yeast assimilable nitrogen (YAN). Organic acids (citric acid, tartaric acid, malic acid, succinic acid, lactic acid, and acetic acid), sugars (glucose, fructose), glycerol and ethanol were determined by HPLC as previously described [23]. The major volatile compounds (acetaldehyde, ethyl acetate, methanol, 1-propanol, 2-methyl-1-propanol [Isobutanol], 3- and 2-methyl-1-butanol) were determined by direct injection of wines into a gas chromatograph according to Nisiotou et al. [23]. The minor volatiles were determined by a headspace SPME/GC-MS method, as described previously [24] with minor modifications [23]. Peaks were quantified relative to internal standards using the peak area of an extracted ion.

### 2.7. Statistical Analysis

Analysis of Variance (ANOVA) and Tukey’s HSD tests were used to determine significant differences between the chemical profiles of wines produced by different inoculation protocols. To investigate the correlations between samples and variables, principal component analysis (PCA) was performed to chemical parameters using the software PRIMER Version 7 (www.primer-e.com, accessed on 30 October 2022). The permutational multivariate analysis of variance (PERMANOVA) was applied to compare between different inoculation protocols. Pairwise distances between inoculation protocols were calculated using the Jaccard metric, and F-statistics were computed using a random sample of 4999 permutations. All tests were conducted with the PAST software (version 3.11) [25].

## 3. Results

### 3.1. Fermentation Kinetics and Yeast Population Dynamics in Mixed Lab-Scale Fermentations 

**Figure 1:**
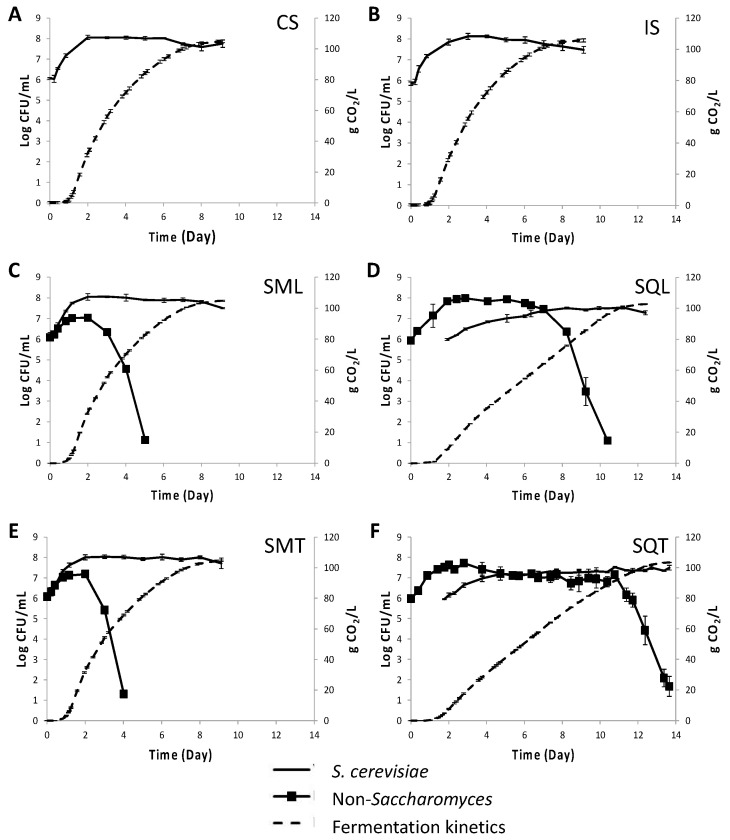
Small-scale laboratory fermentation kinetics (dashed line) and yeast population dynamics (continuous line) in sterile grape must inoculated with (**A**) commercial *S. cerevisiae* (-), (**B**) *S. cerevisiae* ScS13 (-), (**C**) *L. thermotolerans* LtS1 (■) and *S. cerevisiae* ScS13 (-) added simultaneously, (**D**) *L. thermotolerans* LtS1 (■) and *S. cerevisiae* ScS13 (-) added sequentially, (**E**) *T. delbrueckii* TdS6 (■) and *S. cerevisiae* ScS13 (-) added simultaneously, and (**F**) *T. delbrueckii* TdS6 (■) and *S. cerevisiae* ScS13 (-) added sequentially.

The performance of *T. delbrueckii* TdS6, *L. thermotolerans* LtS1 and *S. cerevisiae* ScS13 strains was examined in small-scale fermentations of sterile Assyrtiko grape must under different inoculation schemes, i.e., simultaneous inoculations of ScS13 with TdS6 (SMT) or LtS1 (SML) and sequential inoculations (SQ) of TdS6 (SQT) or LtS1 (SQL) followed by ScS13. Single inoculations with ScS13 (IS) or the commercial *S. cerevisiae* Lalvin QA23 (CS) were also performed as control fermentations. Fermentation kinetics of the various inoculation protocols are shown in Figure 1. The rate of fermentation was lower in SQ than in SM or single-strain inoculations, resulting in a longer fermentation process in SQ (12.4 d in SQL and 13.6 d in SQT) compared to the other inoculation schemes (*ca.* 9 d). The kinetics of *S. cerevisiae* ScS13 were not influenced by the simultaneous addition of either non-*Saccharomyces* (NS) strain (Figure 1). ScS13 reached a plateau at day 2 of fermentation with an average maximum population value (Log CFU/mL) of 7.91 ± 0.09 in IS, 7.95 ± 0.12 in SMT, and 7.89 ± 0.09 in SML. As opposed to this, the kinetics of ScS13 were altered in sequential inoculations. The maximum population of ScS13 in SQ was ca. 0.5 Log CFU/mL lower than in SM or single inoculations, corresponding to maximum average Log CFU/mL values of 7.30 ± 0.09 in SQT and 7.35 ± 0.08 in SQL.

As opposed to ScS13, the NS populations reached higher counts in SQ than in the SM fermentation schemes. More specifically, the maximum population of Tds6 was 0.53 Log CFU/mL higher in SQT than in SMT (i.e., 7.72 ± 0.07 vs. 7.18 ± 0.04, respectively), while the LtS1 population was 0.94 Log CFU/mL higher in SQL than in SML (i.e., 7.98 ± 0.01 vs. 7.04 ± 0.10, respectively). Both NS strains persisted much longer in SQ than in SM fermentations before starting to decline (i.e., 10.8 d in SQT vs. 2.3 d in SMT and 7.0 d in SQL vs. 2.0 d in SML), with Tds6 persisting longer than LtS1 by 3.8 d. Similar death rates were observed for the two NS strains under either SM or SQ inoculation protocol. The populations of both TdS6 and LtS1 declined upon the release of ca. 60 g CO_2_ (corresponding to 8.0% vol ethanol) and 88 g CO_2_ (11.7% vol ethanol), respectively.

### 3.2. Chemical Profile of Laboratory-Scale Ferments

The chemical characteristics and major volatiles of the ferments produced at laboratory-scale fermentations of sterile must are shown in Table 1 and Table 2, respectively. Significant differences were detected in the chemical characteristics of various ferments. The total acidity was significantly higher in SQL compared to other ferments. Concomitantly, SQL exhibited the lowest pH value accompanied by a considerable production of lactic acid. Significant differences were also detected in the levels of volatile acidity, with CS producing the highest levels. Interestingly, the prolonged activity of NS yeasts in SQ inoculations caused a significant drop in volatile acidity, with SQT exhibiting the lowest values. The ferments also showed high variation in major volatiles. Acetaldehyde, ethyl acetate, propanol and isobutanol levels were elevated in SQ fermentations. LtS1 was associated with the highest production of ethyl acetate and propanol. On the other hand, 3-methyl-1-butanol was lower in SQ- than in SM-inoculated ferments.

**Figure 2:**
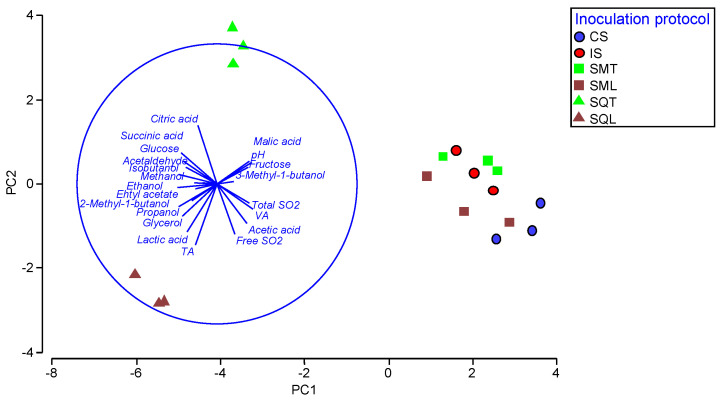
Principal Component Analysis (PCA) of the chemical characteristics of the laboratory-scale produced wines. CS: commercial *S. cerevisiae*; IS: *S. cerevisiae* ScS13; SMT: *T. delbrueckii* TdS6 and *S. cerevisiae* ScS13 added simultaneously; SQT: *T. delbrueckii* TdS6 and *S. cerevisiae* ScS13 added sequentially; SML: *L. thermotolerans* LtS1 and *S. cerevisiae* ScS13 added simultaneously; SQL: *L. thermotolerans* LtS1 and *S. cerevisiae* ScS13 added sequentially.

Overall, the chemical profiles of the ferments were affected by the inoculation protocol (PERMANOVA F = 13.2, *p* < 0.01). Higher F values were observed between SQ and other ferments, exhibiting their high differentiation compared to other treatments. A Principal Component Analysis (PCA) (Figure 2) was applied to depict the similarities between the chemical profiles of the different ferments. The PCA graph represented 54.5% of total variation on PC1and 15.8% on PC2.

CS, IS, SML and SMT ferments were in close proximity, showing high levels of volatile acidity, acetic acid and SO_2_. SQT and SQL formed two well separated groups distantly located on the negative side of the PC1 axis. SQL was differentiated by the highly negative values of lactic acid, total acidity, glycerol and propanol on both PC1 and PC2 axis. SQT showed high positive values on PC2 for succinic acid, acetaldehyde and isobutanol.

### 3.3. Fermentation Kinetics and Yeast Population Dynamics in Mixed Microvinifications

**Figure 3:**
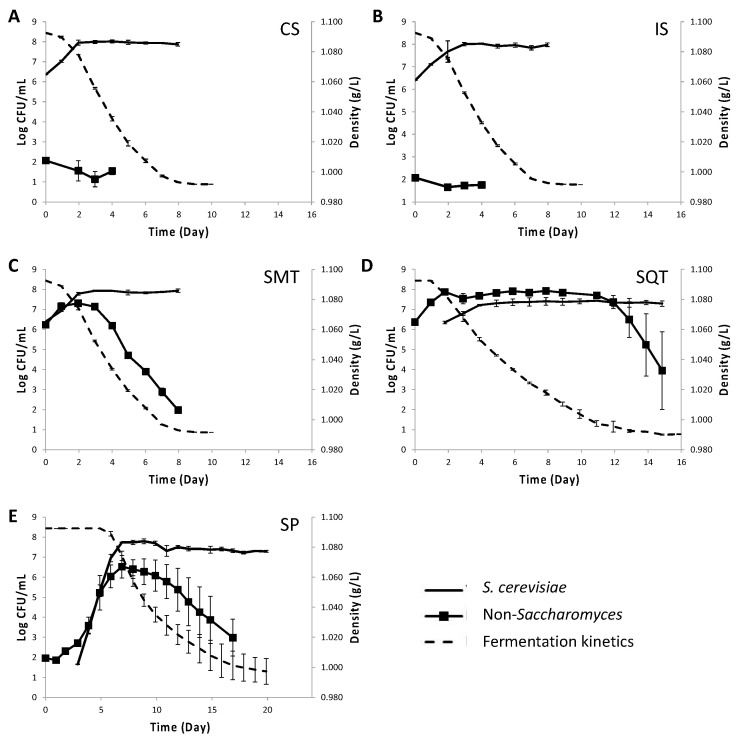
Kinetics of microvinifications (dashed line) and yeast population dynamics (continuous line) in must inoculated with commercial *S. cerevisiae* (**A**), *S. cerevisiae* ScS13 (**B**), simultaneously inoculated *T. delbrueckii* TdS6 and *S. cerevisiae* ScS13 (**C**), sequential inoculated *T. delbrueckii* TdS6 and *S. cerevisiae* ScS13 (**D**) and in non-inoculated must (**E**). ESA medium was used for enumeration of *S. cerevisiae* (-) and LA medium for enumeration of non-*Saccharomyces* yeasts (■).

Based on the above results (i.e., the longer persistence of TdS6 than LtS1 in sequential fermentations and its potential to reduce volatile acidity and acetic acid levels) the strain TdS6 was selected to be used in mini-vinifications of Assyrtiko must along with ScS13 (Figure 3). Similar fermentation kinetics were recorded for the CS (QA23), IS (ScS13), and SM (TdS6 and ScS13) inoculation schemes, where the course was completed by day 9. The fermentation rate in the SQ protocol (TdS6 followed by ScS13) was much lower than the other schemes showing a 6-day delay in the completion of the course. The fermentation rate in spontaneous fermentations (SP) was comparable to SQ until day 11, after which it slowed down leaving residual sugars at the end of the course (Table 2). SP lasted up to day 20, mainly due to its prolonged lag phase. ScS13 showed identical kinetic behavior to the commercial *S. cerevisiae* starter (QA23), with maximum population rising up to 8.01 Log CFU/mL for either strain. The kinetics of ScS13 were unaltered in the SMT scheme, but its maximum population count was lessened by 0.5 Log CFU/mL in SQT (Figure 3C,D). The indigenous *S. cerevisiae* reached higher population levels in SP than in SQ (7.80 ± 0.13 Log CFU/mL) (Figure 3E). ScS13 was the only *S. cerevisiae* strain that was recovered from IS, SM and SQ fermentations, while it was not encountered in SP.

The initial population size of the indigenous NS yeasts in grape must was 2.01 ± 0.02 Log CFU/mL, reaching 6.52 ± 0.57 Log CFU/mL in SP by day 7, while quickly diminishing in CS and IS (Figure 3). In contrast, TdS6 dominated in SM and SQ at maximum population counts of 7.31 ± 0.04 and 7.93 ± 0.12 Log CFU/mL, respectively. Much longer persistence of TdS6 was recorded in SQ (11 d) than in SM (3 d) before its population began to drop. At this point, TdS6 was recovered at 100% frequency from both SM and SQ inoculation schemes.

### 3.4. Chemical Profile of Wines Produced in Microvinifications

The chemical profiles of wines produced in microvinifications are shown in Table 3. The chemical characteristics of the various wines showed significant variations (Table 3). Interestingly, SQT and SP exhibited much higher TA values than the other ferments. The addition of Tds6 significantly reduced the levels of volatile acidity (VA), especially in the SQT inoculation scheme, as compared to IS. In contrast, significantly higher levels of citric acid and succinic acid were recorded in SQT compared to other inoculation schemes. 

Further differences were observed in the volatile profiles of ferments (Table 4). The inoculation of *S. cerevisiae* at high counts at the onset of fermentation was associated with higher levels of esters as compared to either the SQT or SP ferment. In particular, SQT was associated with the lowest levels of esters recorded, diminishing the concentration of ethyl acetate, isoamyl acetate and ethyl octanoate. SQT ferment also exhibited the lowest amount of acetic acid, hexanoic acid and octanoic acid. In contrast, a striking increase was recorded in 2-methyl propanoic acid. Increased levels of linalool, b-citronellol and phenylethyl alcohol were also associated with the SQT inoculation scheme. The highest amounts of terpenes were observed in IS and the lowest respective concentrations in SP.

**Figure 4:**
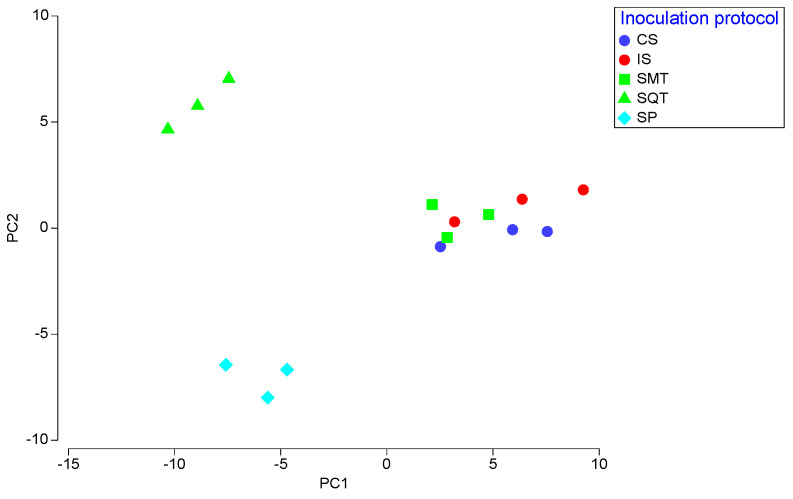
Principal Component Analysis (PCA) of the chemical characteristics wines produced in microvinifications. CS: commercial *S. cerevisiae*; IS: *S. cerevisiae* ScS13; SMT: *T. delbrueckii* TdS6 and *S. cerevisiae* ScS13 added simultaneously; SQT: *T. delbrueckii* TdS6 and *S. cerevisiae* ScS13 added sequentially.

PERMANOVA test indicated significant differences between the inoculation schemes (F = 25.75, *p* < 0.001). SQT ferments exhibited the highest F values in pairwise comparisons with other ferments, revealing high differentiation of SQT ferments from all the other inoculation schemes. The differences between the various inoculation schemes were better visualized on a PCA graph (Figure 4; 50.9% of total variation on PC1 and 21.6% on PC2). CS, IS and SMT ferments were grouped closely to each other, showing positive values on PC1 for numerous characteristics, including damascenone, ethyl acetate, isoamyl acetate and ethyl octanoate. In contrast, SQT and SP were placed on the negative side of the PC1 axis. SQL was well separated from all the other ferments due to highly positive values of 2-methylbutanoic acid, succinic acid and linalool on PC2. SP ferments showed highly negative scores for 2-methyl-1-propanol, methyl esters and residual sugars on both the PC1 and PC2 axes.

### 3.5. Fermentation Kinetics and Yeast Population Dynamics in Mixed Pilot-Scale Fermentations

**Figure 5:**
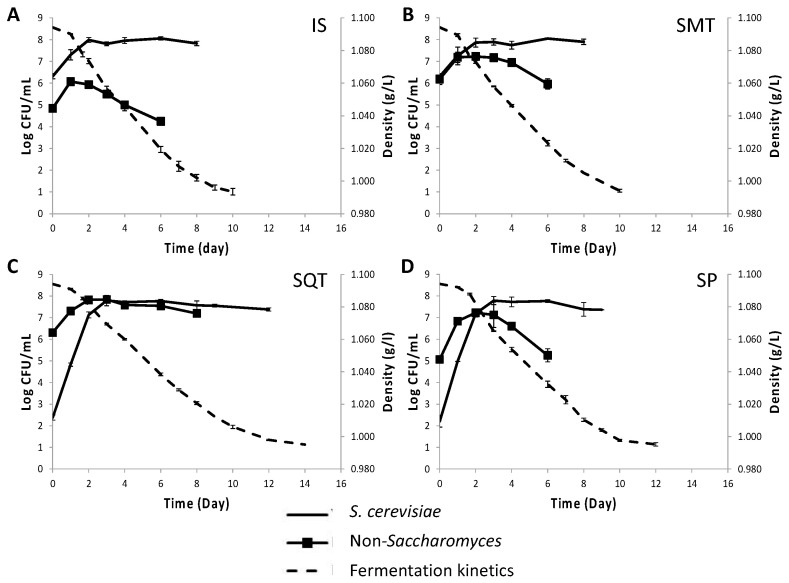
Kinetics of pilot-scale fermentations (dashed line) and yeast populations dynamics (continuous line) in must inoculated with *S. cerevisiae* ScS13 (**A**), simultaneously inoculated *T. delbrueckii* TdS6 and *S. cerevisiae* ScS13 (**B**), sequential inoculated *T. delbrueckii* TdS6 and *S. cerevisiae* ScS13 (**C**) and in non-inoculated must (**D**). ESA medium was used for enumeration for enumeration *S. cerevisiae* (-) and LA medium for non-*Saccharomyces* yeasts (■).

Fermentation kinetics and yeast population dynamics in pilot-scale fermentations with the strains ScS13 and TdS6 are depicted in Figure 5. Similar fermentation dynamics were observed in IS and SMT inoculation schemes, which exhibited the highest fermentation rates resulting in the fastest completion of the course 2 days earlier than in the other treatments. SP showed a lower fermentation rate compared to either IS or SMT but higher than SQT. The kinetic behavior of yeasts varied among the different inoculation schemes. In both IS and SM fermentations, ScS13 followed comparable kinetics, reaching maximum population densities of 8.06 ± 0.08 and 8.05 ± 0.03 Log CFU/mL, respectively (Figure 5A,C). The maximum population count reached by ScS13 was lower in SQT (7.79 ± 0.24 Log CFU/mL) and SP (7.79 ± 0.19 Log CFU/mL) compared to those in IS and SMT fermentations (Figure 5B,D). ScS13 prevailed in both IS and SM fermentations at percentages of 100% and 86–100%, respectively. The respective percentages were lower in SQT fermentations (23–35%) in which two indigenous yeast populations also evolved. The ScS13 strain was not detected in SP fermentations.

Significant differences were observed in the non-*Saccharomyces* (NS) yeast kinetics among the different inoculation protocols applied. In the IS scheme, the indigenous NS yeast populations, which were at 4.96 ± 0.16 Log CFU/mL, were quickly suppressed by the early addition of ScS13 reaching a maximum population of 6.07 ± 0.13 Log CFU/mL by day 1, and thereafter started to decline sharply. In contrast, in SP the NS yeast population reached higher counts (7.23 ± 0.07 Log CFU/mL) and had longer persistence. The addition of TdS6 increased the initial counts in LA medium by more than 1 Log CFU/mL in both SMT and SQT as compared to IS and SP ferments (Figure 5). In SM ferments, the NS counts peaked at day 1 at higher average values (7.19 ± 0.09 Log CFU/mL) than in either IS (6.00 ± 0.05 Log CFU/mL). In SP, NS counts peaked at 7.06 ± 0.30 Log CFU/mL Significantly higher NS counts (7.84 ± 0.04 Log CFU/mL) and also persistence were recorded in SQT compared to the other protocols (Figure 5). At day 6 of the alcoholic fermentation, the strain TdS6 was isolated at frequencies of 100% and 60% from LA agar in SQT and SMT ferments, respectively.

### 3.6. Chemical Profile of Wines Produced in Pilot-Scale Fermentations

The chemical characteristics of wines produced at pilot scale are presented in Table 5. The most striking differences were observed in the levels of VA and acetic acid that were lower in SQT compared to the other ferments. On the other hand, the succinic acid concentration was significantly higher in SQT than in other wines. Concerning the major volatile constituents of wines, the use of TdS6 caused a significant increase in the levels of isobutanol, 2-methyl-1-butanol and 3-methyl-1-butanol (Table 6). Significant differences were also recorded in the minor volatile profiles of wines (Table 7). The addition of TdS6 reduced the levels of esters, similarly to the laboratory-scale fermentations. The decline in ester concentrations was most prominent in SQT, showing high decreases in the levels of ethyl acetate, isoamyl acetate, hexyl acetate and ethyl octanoate. Overall, despite differences that were not as pronounced, the chemical profiles of wines made using various inoculation protocols in pilot-scale fermentations followed the same trends as in laboratory-scale fermentations.

**Figure 6:**
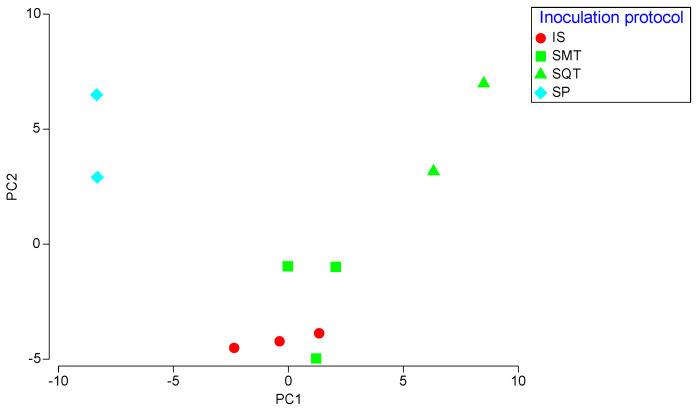
Principal Component Analysis (PCA) of the chemical characteristics of the pilot-scale produced wines. IS: *S. cerevisiae* ScS13; SMT: *T. delbrueckii* TdS6 and *S. cerevisiae* ScS13 added simultaneously; SQL: *T. delbrueckii* TdS6 and *S. cerevisiae* ScS13 added sequentially; SP: spontaneous fermentation.

Significant variations between the chemical profiles of the different wines were observed by PERMANOVA (F = 3.27, *p* = 0.001). A PCA graph was constructed to better depict chemical variation of the wines produced at pilot-scale fermentation (Figure 6; 26.8% of total variation on PC1 and 20.2% on PC2). IS and SMT ferments were grouped closely to each other on the negative side of PC2. IS was characterized by highly negative values of 3-methyl pentanol and 4-methyl pentanol while SMT of citronellol and ethyl hexanol. The SQT ferments were located on the positive side of PC1 with high positive values for several metabolites, including nerolidol and citric acid. SP formed a separate cluster scoring positively on PC2 for several volatiles, such as ethyl acetate and acetic acid.

## 4. Discussion

‘Assyrtiko’ is one of the greatest white Greek grape varieties originated from the island of Santorini in the Aegean Sea. It is most often used to produce high quality dry white wines of complex texture, body and structure rather than aromatic style wines. Assyrtiko-Santorini is highly correlated with the volcanic terroir of Santorini, its original place of origin, where lean, highly acidic and less aromatic but mineral and very concentrated wines are produced. When cultivated in other regions, Assyrtiko wines tend to be less dense in structure or minerality. Due to the detrimental effects of global warming on diminishing wine acidity in warm regions, the unique and distinctive terroir of Assyrtiko-Santorini is currently under threat. To increase the acidity while boosting the terroir expression of this emblematic volcanic wine, two indigenous NS yeast species with promising technological characteristics along with an indigenous *S. cerevisiae* strain were investigated in various fermentation volumes and inoculation schemes.

*S. cerevisiae* is known to be highly competitive against NS yeasts and often outcompetes their population when added at the onset of the fermentation course. Accordingly, in this study, both NS yeasts tested quickly declined when co-inoculated with *S. cerevisiae*. The drop of NS yeasts at early addition of *S. cerevisiae* has been attributed to the increased sugar fermentation ability and nitrogen absorption of *S. cerevisiae* compared to NS wine yeasts [26,27]. In sequential fermentations, however, both NS yeasts exhibited an extended persistence with *T. delbrueckii* persisting the longest. The prolonged viability of both *S. cerevisiae* and *T. delbrueckii* when sequentially inoculated in grape must has been observed previously [28]. According to Álvarez-Fernández et al. [29], since *T. delbrueckii* utilizes nitrogen through different metabolic pathways than *S. cerevisiae*, it may be easier for both species to coexist in mixed fermentations for a longer period of time. Therefore, *T. delbrueckii* strains may exhibit good compatibility with *S. cerevisiae* in mixed fermentations, a fact that may facilitate their use as yeast starter cultures in commercial applications. However, the fermentative power of each particular *T. delbrueckii* strain needs to be carefully considered since it appears to be highly strain-dependent, with evidence supporting the existence of strains showing either good [15,16,30] or poor fermentation performance [31,32,33,34].

In the present study it was shown that the sequential inoculation scheme with either *L. thermotolerans* LtS1 or *T. delbrueckii* TdS6 had greater differentiating effect as compared to simultaneous co-inoculation on the wine chemical profile in small-scale laboratory fermentations. The use of *L. thermotolerans* caused a significant rise in the titratable acidity (TA) of the ferment driven by the high production of lactic acid. *L. thermotolerans* is well known for its capacity to produce elevated levels of lactic acid and for this reason it has been considered as a powerful means to achieve biological acidification in wines [11,35]. Although to a lesser extent, *T. delbrueckii* TdS6 also showed the ability to increase TA through the production of succinic acid. Furthermore, the reduced levels of volatile acidity (VA) observed in sequential inoculations with *T. delbrueckii* is of high importance in fermentation of Assyrtiko grape must, which is often characterized by high sugar content and elevated VA levels. The use of a low-VA producing strain, such as *T. delbrueckii* TdS6 here, would significantly ameliorate the VA levels of ‘Visanto’, a prestigious naturally sweet wine of Santorini, which is mostly made from Assyrtiko must. The use of several NS strains, mainly of *Starmerella bacillaris*, in wine fermentation has been recently considered as a strategy to counteract the elevated acetic acid production mostly at the beginning of fermentation by *S. cerevisiae* [23,36,37]. Among other yeasts, *T. delbrueckii* has been shown previously to generate reduced levels of acetic acid, even during fermentation at very high sugar concentrations [38]. In the present study, *T. delbrueckii* showed much higher potential to reduce VA than *L. thermotolerans* LtS1. The strain TdS6 was further shown to largely decrease both VA and acetic acid levels in pilot-scale production of wine.

On the other hand, *T. delbrueckii* significantly increased the levels of succinic acid in wine. This elevation of succinic acid levels has been proposed to be coupled with acetic acid decline, as described above. Accordingly, it has been suggested that a decrease in acetic acid redirects carbon flux to the overproduction of other metabolites, such as succinate [39]. Although succinic acid is the second most abundant non-volatile by-product of alcoholic fermentation [40], its relevance in wine is sometimes neglected due to its low acidity. Yet, succinic acid is considered to be the principal driver of titratable acidity rises in the finished product. Added to this, significant increases in the TA were observed in wines with high succinic acid production in this study. Taken together, succinate-producing *T. delbrueckii* strains may be considered as a significant candidate NS yeast starter for biological acidification of must. This biological activity is of particular interest in the wine industry to address low acidity in wines, an emerging threat in the grape/wine sector due to global warming [9].

Succinic acid has also a very important sensorial role. Besides increasing sourness, it brings salty and bitter tastes in wine [41]. High concentrations of succinic acid were associated with increased perceived minerality in white wines [42]. Therefore, the use of *T. delbrueckii* could highlight the mineral notes and enhance the perceived salinity that characterize Assyrtiko wines. Furthermore, succinic acid is also a precursor for aroma-active esters which bring mild and fruity aromas in wines, such as diethyl succinate in aged wines or sherry wines [43].

Ester formation is a key qualitative characteristic of yeast activity, adding pleasant notes to wine aroma [44]. In the present study, diethyl succinate (fruity), ethyl propanoate (pineapple) and ethyl isobutyrate (sweet, fruity), which are considered to be activity markers of *T. delbrueckii* [45], were significantly increased in mixed sequential inoculations. Some other ethyl esters were also increased, such as ethyl heptanoate (fruity, apple, grape) [46], ethyl lactate (fruity, buttery and creamy aromas, with sense of roundness in the mouth) [47,48], and ethyl 2-methylbutanoate. Despite the increase in the aforementioned esters, a decline in the net total ester content was observed in *T. delbrueckii*-inoculated sequential fermentations. More precisely, the use of TdS6 lowered the concentration of important esters in wine, such as ethyl acetate, isoamyl acetate or ethyl octanoate. A lower ester content in mixed fermentations with *T. delbrueckii* as compared to single-inoculation with *S. cerevisiae* has been also reported previously [37,49]. In some other cases, however, the use of *T. delbrueckii* in mixed fermentations with *S. cerevisiae* was shown to increase the total ester concentration [32,45,50]. A plausible explanation for those divergent outcomes is the high intra-species diversity of *T. delbrueckii* [51]. In fact, accumulating data reveal a significant diversity in the enological phenotypes among *T. delbrueckii* isolates [15].

In conclusion, the present results show that the use of indigenous *S. cerevisiae* in mixed culture with *L. thermotolerans* or *T. delbrueckii* strains may introduce significant diversification of the wines compared to the use of a commercial starter. *T. delbrueckii* TdS6 in particular showed several important technological characteristics that may be of certain usefulness in the production of Assyrtiko-Santorini wine. Among others, the capacity of TdS6 to diminish VA and to increase TA coupled with good compatibility with *S. cerevisiae* dictates its use in the fermentation of high sugar musts for the production of structured dry or sweet wines rather than aromatic style wines.

## Figures and Tables

**Table 1:** Chemical parameters of ferments produced in laboratory-scale fermentations of sterile must (mean ± SD, *n* = 3). Different letters indicate significantly different values (*p* < 0.05).

Chemical Parameter	Inoculation Protocol ^1^					
	CS	IS	SML	SQL	SMT	SQT
Total acidity (as tartaric acid g/L)	9.9 ± 0.1 ^b^	9.9 ± 0.1 ^b^	10.0 ± 0.0 ^b^	11.6 ± 0.1 ^a^	9.9 ± 0.0 ^b^	9.4 ± 0.1 ^c^
pH	3.23 ± 0.01 ^b^	3.25 ± 0.01 ^a^	3.20 ± 0.01 ^c^	3.16 ± 0.01 ^d^	3.26 ± 0.01 a	3.20 ± 0.01 ^c^
Volatile acidity (as acetic acid g/L)	0.43 ± 0.01 ^a^	0.34 ± 0.01 ^b^	0.39 ± 0.02 ^c^	0.18 ± 0.01 ^d^	0.30 ± 0.01 ^e^	0.12 ± 0.01 ^f^
Free SO2 (mg/L)	11.5 ± 2.5 ^a^	9.7 ± 1.9 ^ab^	11.4 ± 1.4 ^a^	9.8 ± 0.7 ^ab^	9.0 ± 0.0 ^ab^	6.8 ± 2.0 ^b^
Total SO2 (mg/L)	24.7 ± 1.5 ^a^	19.2 ± 0.0 ^cd^	23.9 ± 0.8 ^ab^	17.1 ± 0.7 ^d^	21.3 ± 1.4 ^bc^	17.1 ± 0.8 ^d^
Citric acid (mg/L)	359 ± 6 ^a^	365 ± 1 ^a^	363 ± 5 ^a^	361 ± 1 ^a^	359 ± 6 ^a^	397 ± 3 ^b^
Tartaric acid (g/L)	4.7 ± 0.1 ^c^	4.8 ± 0.0 ^ab^	4.8 ± 0.0 ^ab^	4.9 ± 0.0 ^a^	4.8 ± 0.0 ^bc^	4.8 ± 0.0 ^abc^
Malic acid (g/L)	2.0 ± 0.0 ^c^	2.1 ± 0.0 ^a^	2.0 ± 0.0 ^bc^	1.8 ± 0.0 ^e^	2.0 ± 0.0 ^ab^	1.9 ± 0.0 ^d^
Glucose (g/L)	<0.6	<0.6	<0.6	<0.6	<0.6	<0.6
Fructose (g/L)	1.1 ± 0.0 ^c^	1.6 ± 0.1 ^a^	1.4 ± 0.1 ^b^	0.8 ± 0.1 ^d^	1.6 ± 0.1 ^a^	1.0 ± 0.1 ^c^
Succinic acid (g/L)	0.7 ± 0.0 ^d^	0.7 ± 0.0 ^cd^	0.7 ± 0.0 ^c^	0.8 ± 0.0 ^b^	0.7 ± 0.0 ^c^	0.8 ± 0.0 ^a^
Lactic acid (g/L)	<0.6	<0.6	<0.6	3.1 ± 0.1	<0.6	<0.6
Glycerol (g/L)	6.0 ± 0.1 ^c^	6.1 ± 0.0 ^bc^	6.2 ± 0.1 ^bc^	6.7 ± 0.0 ^a^	6.1 ± 0.1 ^bc^	6.2 ± 0.1 ^b^
Acetic acid (mg/L)	298 ± 18 ^a^	241 ± 8 ^b^	182 ± 33 ^c^	156 ± 5 ^c^	187 ± 4 ^c^	69 ± 6 ^d^
Ethanol (g/L)	105.7 ± 1.8 ^a^	107.0 ± 0.6 ^a^	106.5 ± 1.7 ^a^	106.8 ± 0.1 ^a^	105.6 ± 1.3 ^a^	106.7 ± 1.6 ^a^

^1^ CS: commercial *S. cerevisiae*; IS: *S. cerevisiae* ScS13; SML: *L. thermotolerans* LtS1 and *S. cerevisiae* ScS13 added simultaneously; SQL: *L. thermotolerans* LtS1 and *S. cerevisiae* ScS13 added sequentially; SMT: *T. delbrueckii* TdS6 and *S. cerevisiae* ScS13 added simultaneously; SQT: *T. delbrueckii* TdS6 and *S. cerevisiae* ScS13 added sequentially.

**Table 2:** Major volatiles (mg/L) of ferments produced in laboratory-scale fermentations of sterile must (mean ± SD, *n* = 3). Different letters indicate significantly different values (*p* < 0.05).

Chemical Parameter	Inoculation Protocol ^1^					
	CS	IS	SML	SQL	SMT	SQT
Acetaldehyde	7.0 ± 0.8 ^ab^	6.5 ± 0.4 ^b^	7.4 ± 1.7 ^ab^	8.4 ± 0.5 ^ab^	6.7 ± 0.3 ^ab^	8.9 ± 0.2 ^a^
Ethyl acetate	58.1 ± 2.3 ^c^	59.4 ± 1.7 ^c^	62.7 ± 2.7 ^c^	159.0 ± 3.1 ^a^	62.0 ± 1.1 ^c^	131.4 ± 4.5 ^b^
Methanol	46.4 ± 1.3 ^a^	47.5 ± 1.4 ^a^	46.3 ± 1.1 ^a^	48.8 ± 1.3 ^a^	47.7 ± 1.3 ^a^	47.8 ± 0.7 ^a^
Propanol	31.7 ± 0.7 ^c^	30.5 ± 0.6 ^c^	30.9 ± 0.5 ^c^	97.6 ± 2.2 ^a^	30.9 ± 0.4 ^c^	59.3 ± 1.9 ^b^
Isobutanol	25.2 ± 1.4 ^b^	25.0 ± 0.6 ^b^	24.4 ± 0.7 ^b^	35.4 ± 0.3 ^a^	23.3 ± 0.5 ^b^	35.7 ± 1.8 ^a^
2-Methyl-1-butanol	21.4 ± 2.4 ^b^	23.4 ± 0.7 ^ab^	24.6 ± 0.3 ^a^	25.4 ± 0.4 ^a^	24.6 ± 0.7 ^a^	24.1 ± 1.0 ^ab^
3-Methyl-1-butanol	122.4 ± 1.3 ^c^	134.4 ± 5.1 ^ab^	141.5 ± 0.7 ^a^	125.6 ± 2.3 ^bc^	140.7 ± 3.9 ^a^	123.8 ± 7.3 ^bc^

^1^ CS: commercial *S. cerevisiae*; IS: *S. cerevisiae* ScS13; SML: *L. thermotolerans* LtS1 and *S. cerevisiae* ScS13 added simultaneously; SQL: *L. thermotolerans* LtS1 and *S. cerevisiae* ScS13 added sequentially; SMT: *T. delbrueckii* TdS6 and *S. cerevisiae* ScS13 added simultaneously; SQT: *T. delbrueckii* TdS6 and *S. cerevisiae* ScS13 added sequentially.

**Table 3:** Chemical parameters of wines produced in microvinifications (mean ± SD, *n* = 3). Values not sharing a letter are significantly different (*p* < 0.05).

Chemical Parameter	Inoculation Protocol ^1^				
	CS	IS	SMT	SQT	SP
Total acidity (as tartaric acid g/L)	9.6 ± 0.1 ^b^	9.3 ± 0.1 ^b^	9.2 ± 0.1 ^b^	10.4 ± 0.2 ^a^	10.6 ± 0.4 ^a^
pH	2.95 ± 0.01 ^c^	2.97 ± 0.01 ^bc^	2.97 ± 0.00 ^b^	3.03 ± 0.01 ^a^	2.90 ± 0.01 ^d^
Volatile acidity (as acetic acid g/L)	0.96 ± 0.04 ^ab^	0.86 ± 0.04 ^bc^	0.64 ± 0.03 ^cd^	0.37 ± 0.12 ^d^	1.22 ± 0.21 ^a^
Ethanol (vol%)	13.08 ± 0.02 ^a^	12.93 ± 0.07 ^ab^	12.90 ± 0.04 ^ab^	12.84 ± 0.05 ^ab^	12.47 ± 0.41 ^b^
Glucose (g/L)	<0.6	<0.6	<0.6	<0.6	0.8 ± 1.0
Fructose (g/L	0.9 ± 0.1 ^a^	0.8 ± 0.1 ^a^	0.7 ± 0.1 ^a^	<0.6	7.4 ± 7.4 ^a^
Citric acid (mg/L)	460 ± 9 ^d^	470 ± 4 ^d^	497 ± 2 ^c^	632 ± 13 ^a^	569 ± 10 ^b^
Tartaric acid (g/L)	5.8 ± 0.1 ^ab^	5.7 ± 0.2 ^b^	5.7 ± 0.1 ^b^	6.0 ± 0.1 ^a^	6.0 ± 0.0 ^a^
Malic acid (g/L)	1.7 ± 0.0 ^a^	1.7 ± 0.0 ^a^	1.7 ± 0.0 ^a^	1.7 ± 0.1 ^a^	1.7 ± 0.1 ^a^
Succinic acid (g/L)	<0.6	<0.6	<0.6	1.0 ± 0.0 ^a^	0.6 ± 0.1 ^b^
Lactic acid (g/L)	<0.6	<0.6	<0.6	<0.6	<0.6
Glycerol (g/L)	7.4 ± 0.1 ^a^	7.2 ± 0.0 ^a^	7.0 ± 0.0 ^a^	6.2 ± 0.1 ^b^	6.7 ± 0.6 ^ab^
Ethanol (g/L)	105.5 ± 0.2 ^a^	105.7 ± 0.2 ^a^	106.0 ± 0.0 ^a^	105.8 ± 0.3 ^a^	101.0 ± 2.8 ^b^

^1^ CS: commercial *S. cerevisiae*; IS: *S. cerevisiae* ScS13; SMT: *T. delbrueckii* TdS6 and *S. cerevisiae* ScS13 added simultaneously; SQT: *T. delbrueckii* TdS6 and *S. cerevisiae* ScS13 added sequentially. SP: Spontaneous fermentation.

**Table 4:** Minor volatiles (μg/L) ^1^ of wines produced in microvinifications (mean ± SD, *n* = 3 or *n* = 2 in SP). Values not sharing a letter are significantly different (*p* < 0.05).

Chemical Component	Inoculation Protocol ^2^				
	CS	IS	SMT	SQT	SP
**Esters**					
Methyl acetate	3 ± 1 ^a^	3 ± 1 ^ab^	3 ± 1 ^ab^	1 ± 0 ^b^	4 ± 1 ^a^
Ethyl acetate	92,310 ± 9364 ^a^	98,640 ± 16,693 ^a^	84,633 ± 12,904 ^a^	40,757 ± 1513 ^b^	67,993 ± 16,135 ^ab^
Propyl acetate	429 ± 91 ^b^	655 ± 111 ^a^	537 ± 35 ^ab^	105 ± 52 ^c^	126 ± 64 ^c^
Butyl acetate	95 ± 21 ^a^	104 ± 14 ^a^	95 ± 7 ^a^	7 ± 12 ^b^	8 ± 14 ^b^
Isobutyl acetate	76,403 ± 17,864 ^ab^	91,863 ± 14,120 ^a^	75,240 ± 4668 ^ab^	32,517 ± 9707 ^c^	49,240 ± 16,881 ^bc^
Isoamyl acetate	3,156,060 ± 794,922 ^a^	3,959,940 ± 513,096 ^a^	3,085,093 ± 159,870 ^a^	683,323 ± 341,193 ^b^	1,013,410 ± 315,672 ^b^
Hexyl acetate	176,437 ± 27,004 ^a^	193,663 ± 19,067 ^a^	153,960 ± 1892 ^a^	76,237 ± 5435 ^b^	99,857 ± 15,010 ^b^
(3E)-3-Hexenyl acetate	158 ± 33 ^ab^	192 ± 30 ^a^	132 ± 8 ^b^	14 ± 7 ^c^	31 ± 10 ^c^
(3Z)-3-Hexenyl acetate	70 ± 17 ^ab^	86 ± 14 ^a^	51 ± 2 ^b^	2 ± 4 ^c^	22 ± 6 ^c^
Octyl acetate	51 ± 11 ^a^	44 ± 10 ^ab^	29 ± 2 ^b^	0 ± 0 ^c^	0 ± 0 ^c^
2-Phenylethyl acetate	586,283 ± 17,186 ^ab^	896,080 ± 24,956 ^a^	861,183 ± 129,492 ^a^	277,427 ± 64,550 ^b^	728,957 ± 216,781 ^a^
Ethyl butanoate	382,167 ± 56,878 ^a^	415,743 ± 43,924 ^a^	365,343 ± 16,545 ^a^	231,670 ± 47,209 ^b^	24,2107 ± 36,357 ^b^
Ethyl 2-methylbutanoate	4 ± 4 ^b^	14 ± 7 ^ab^	13 ± 7 ^ab^	23 ± 7 ^a^	11 ± 6 ^ab^
Ethyl 3-methylbutanoate	27 ± 8 ^a^	34 ± 6 ^a^	25 ± 1 ^a^	20 ± 8 ^a^	17 ± 7 ^a^
Ethyl isobutyrate	89,027 ± 2772 ^b^	91,033 ± 2407 ^b^	90,987 ± 774 ^b^	109,867 ± 12,331 ^a^	95,800 ± 7802 ^ab^
Methyl hexanoate	188 ± 44 ^ab^	197 ± 16 ^a^	154 ± 13 ^abc^	95 ± 19 ^c^	121 ± 31 ^bc^
Ethyl hexanoate	719,013 ± 164,912 ^a^	661,823 ± 103,961 ^a^	498,310 ± 15,558 ^ab^	142,603 ± 40,088 ^c^	312,660 ± 90,314 ^bc^
Methyl heptanoate	6 ± 1 b	5 ± 0 ^b^	5 ± 1 ^b^	5 ± 0 ^b^	10 ± 2 ^a^
Ethyl lactate	3037 ± 267 ^b^	3623 ± 556 ^b^	3570 ± 167 ^b^	5890 ± 1536 ^a^	4317 ± 775 ^ab^
Methyl octanoate	24 ± 5 ^b^	20 ± 1 ^b^	19 ± 5 ^b^	19 ± 1 ^b^	40 ± 7 ^a^
Ethyl octanoate	1,022,913 ± 250,174 ^a^	904,203 ± 233,561 ^ab^	715,090 ± 39,805 ^ab^	156,007 ± 42,462 ^c^	490,823 ± 139,919 ^bc^
Isoamyl hexanoate	82 ± 20 ^a^	70 ± 25 ^ab^	67 ± 4 ^ab^	5 ± 8 ^c^	31 ± 11 ^bc^
Methyl nonanoate	19 ± 3 ^ab^	18 ± 1 ^b^	18 ± 3 ^b^	19 ± 0 ^b^	26 ± 5 ^a^
Methyl decanoate	11 ± 1 ^b^	10 ± 0 ^b^	11 ± 1 ^b^	10 ± 1 ^b^	14 ± 1 ^a^
Ethyl decanoate	464,663 ± 120,974 ^a^	395,470 ± 105,413 ^a^	469,690 ± 6,2521 ^a^	44,420 ± 14,272 ^b^	262,493 ± 124,231 ^ab^
3-Methylbutyl octanoate	446 ± 91 ^a^	382 ± 113 ^a^	502 ± 106 ^a^	60 ± 22 ^b^	329 ± 173 ^ab^
Diethyl butanedioate	22,930 ± 871 ^c^	25,420 ± 859 ^bc^	25,917 ± 1690 ^bc^	40,740 ± 2002 ^b^	69,097 ± 12,591 ^a^
Ethyl 9-decenoate	17,769 ± 3519 ^a^	16,565 ± 4162 ^a^	13,494 ± 622 ^a^	2064 ± 531 ^b^	19,278 ± 7671 ^a^
Ethyl dodecanoate	2829 ± 263 ^bc^	2756 ± 505 ^bc^	6360 ± 1374 ^a^	628 ± 199 ^c^	4923 ± 2294 ^ab^
**Alcohols**					
1-Propanol	58,650 ± 4675 ^ab^	65,740 ± 11,472 ^a^	67,137 ± 5766 ^a^	41,390 ± 11,069 ^bc^	33,770 ± 9909 ^c^
2-Methyl-1-propanol	26,320 ± 1508 ^a^	25,087 ± 3592 ^a^	26,327 ± 1007 ^a^	24,047 ± 6316 ^a^	32,677 ± 6100 ^a^
3-(Methylthio)-1-propanol	1 ± 0 ^c^	2 ± 0 ^c^	2 ± 0 ^bc^	6 ± 1 ^a^	4 ± 1 ^b^
1-Butanol	39 ± 2 ^a^	37 ± 5 ^a^	41 ± 1 ^a^	28 ± 8 ^a^	35 ± 8 ^a^
3-Methyl-1-butanol	166,333 ± 27,191 ^a^	142,990 ± 23,921 ^ab^	132,873 ± 19,068 ^ab^	107,833 ± 40,643 ^ab^	76,623 ± 16,431 ^b^
4-Methyl-1-pentanol	3 ± 0 ^b^	5 ± 0 ^a^	5 ± 0 ^a^	3 ± 1 ^b^	3 ± 0 ^b^
3-Methyl-1-pentanol	7 ± 0 ^bc^	9 ± 0 ^ab^	10 ± 1 ^a^	6 ± 1 ^bc^	8 ± 1 ^bc^
1-Hexanol	397,970 ± 13,441 ^b^	341,490 ± 13,450 ^b^	359,817 ± 34,917 ^b^	536,787 ± 17,438 ^a^	488,857 ± 20,199 ^a^
(E)-3-Hexen-1-ol	2 ± 0 ^a^	2 ± 0 ^a^	2 ± 0 ^a^	3 ± 0 ^a^	1 ± 0 ^b^
2-Ethyl-1-hexanol	52,530 ± 684 ^a^	52,003 ± 208 ^a^	52,033 ± 81 ^a^	52,990 ± 936 ^a^	52,287 ± 499 ^a^
2,3-Butanediol (isomer 1)	311 ± 17 ^a^	254 ± 38 ^a^	225 ± 48 ^a^	87 ± 3 ^b^	237 ± 41 ^a^
2,3-Butanediol (isomer 2)	71 ± 6 ^a^	57 ± 9 ^ab^	53 ± 10 ^ab^	29 ± 2 ^c^	49 ± 8 ^bc^
1-Heptanol	11 ± 0 ^b^	10 ± 0 ^b^	10 ± 0 ^b^	11 ± 3 ^b^	28 ± 6 ^a^
1-Octanol	1437 ± 76 ^a^	1257 ± 32 ^ab^	1207 ± 85 ^ab^	827 ± 81 ^c^	1040 ± 135 ^bc^
1-Octen-3-ol	0 ± 1 ^a^	1 ± 1 ^a^	1 ± 0 ^a^	1 ± 1 ^a^	1 ± 0 ^a^
1-Decanol	2 ± 0 ^ab^	2 ± 0 ^ab^	2 ± 0 ^b^	2 ± 0 ^a^	2 ± 0 ^ab^
1-Nonanol	91 ± 25 ^a^	87 ± 21 ^a^	64 ± 2 ^a^	79 ± 18 ^a^	80 ± 34 ^a^
Phenylethyl Alcohol	23,130 ± 1898 ^a^	26,287 ± 3234 ^a^	26,723 ± 1466 ^a^	29,847 ± 6030 ^a^	20,823 ± 2383 ^a^
**Acids**					
Acetic acid	38,684 ± 5425 ^a^	35,160 ± 8404 ^a^	27,271 ± 2660 ^ab^	12,116 ± 785 ^b^	33,209 ± 10,457 ^a^
2-Methylpropanoic acid	313 ± 27 ^b^	315 ± 46 ^b^	376 ± 50 ^b^	1376 ± 214 ^a^	619 ± 144 ^b^
Butanoic acid	401 ± 41 ^a^	408 ± 67 ^a^	374 ± 96 ^a^	374 ± 39 ^a^	334 ± 69 ^a^
2-Methylbutanoic acid	114 ± 8 ^b^	140 ± 20 ^b^	138 ± 18 ^b^	282 ± 82 ^a^	101 ± 25 ^b^
3-Methylbutanoic acid	372 ± 27 ^a^	335 ± 76 ^a^	319 ± 16 ^a^	374 ± 71 ^a^	270 ± 85 ^a^
Hexanoic acid	1,790,940 ± 108,791 ^a^	1,537,050 ± 209,891 ^ab^	1,332,070 ± 128,762 ^b^	395,363 ± 12,067 ^c^	1,264,727 ± 165,953 ^b^
Octanoic acid	10,728 ± 1484 ^a^	10,341 ± 884 ^a^	8993 ± 1302 ^ab^	2318 ± 271 ^c^	6418 ± 284 ^b^
n-Decanoic acid	2271 ± 523 ^a^	2500 ± 293 ^a^	2278 ± 372 ^a^	491 ± 118 ^b^	895 ± 119 ^b^
**Carbonyl compounds**					
Acetaldehyde	47 ± 7 ^a^	67 ± 17 ^a^	87 ± 28 ^a^	47 ± 7 ^a^	89 ± 39 ^a^
2-Octanone	906 ± 191 ^a^	957 ± 118 ^a^	736 ± 37 ^ab^	541 ± 105 ^b^	528 ± 104 ^b^
2-Nonanone	6 ± 1 ^a^	6 ± 1 ^a^	5 ± 0 ^a^	0 ± 1 ^b^	0 ± 0 ^b^
2,3-Butanedione	27 ± 4 ^ab^	34 ± 7 ^a^	28 ± 1 ^a^	13 ± 5 ^c^	15 ± 5 ^bc^
**Terpenes**					
b-Citronellol	5957 ± 520 ^a^	4957 ± 150 ^b^	4717 ± 166 ^b^	6130 ± 148 ^a^	3747 ± 176 ^c^
Linalool	63,233 ± 940 ^ab^	62,843 ± 456 ^ab^	62,953 ± 1140 ^ab^	65,083 ± 1168 ^a^	61,017 ± 762 ^b^
Eucalyptol	13 ± 2 ^a^	15 ± 4 ^a^	12 ± 1 ^a^	10 ± 2 ^a^	10 ± 1 ^a^
a-Terpineol	30,970 ± 2905 ^a^	36,530 ± 7206 ^a^	28,957 ± 9618 ^a^	22,430 ± 2737 ^ab^	10,193 ± 880 ^b^
b-Damascenone	200 ± 6 ^a^	192 ± 1 ^a^	177 ± 20 ^a^	111 ± 5 b	97 ± 15 ^b^
a-Pinene	39,243 ± 860 ^ab^	40,053 ± 851 ^a^	39,270 ± 949 ^ab^	37,943 ± 447 ^b^	37,453 ± 310 ^b^
Terpene_1095	157 ± 40 ^ab^	206 ± 46 ^a^	160 ± 44 ^ab^	105 ± 24 ^b^	71 ± 24 ^b^
α- Phellandrene	241 ± 59 ^ab^	296 ± 56 ^a^	228 ± 65 ^ab^	157 ± 27 ^bc^	89 ± 13 ^c^
b-Myrcene	186 ± 46 ^ab^	220 ± 40 ^a^	169 ± 48 ^ab^	122 ± 21 ^bc^	65 ± 12 ^c^
D-Limonene	38,513 ± 9672 ^ab^	45,867 ± 8264 ^a^	34,487 ± 9112 ^ab^	25,373 ± 4260 ^bc^	13,853 ± 2179 ^c^
gamma-Terpinene	24,660 ± 1324 ^ab^	25,523 ± 1010 ^a^	23,977 ± 1149 ^ab^	22,763 ± 519 ^bc^	21,403 ± 191 ^c^
p-Cymene	15,857 ± 1058 ^a^	15,823 ± 596 ^a^	15,380 ± 1099 ^a^	14,087 ± 279 ^ab^	13,330 ± 135 ^b^
**Other compounds**					
1,1-Diethoxy ethane	42 ± 6 ^ab^	47 ± 6 ^ab^	40 ± 6 ^b^	49 ± 2 ^ab^	93 ± 42 ^a^
Naphthalene	161 ± 18 ^a^	179 ± 19 ^a^	142 ± 10 ^ab^	108 ± 11 ^bc^	91 ± 16 ^c^

^1^ Concentrations relative to internal standard (3-pentanol). Expressed as the ratio of each compound peak area to that of internal standard multiplied by its concentration (1000 μg/L). ^2^ CS: commercial *S. cerevisiae*; IS: *S. cerevisiae* ScS13; SMT: *T. delbrueckii* TdS6 and *S. cerevisiae* ScS13 added simultaneously; SQT: *T. delbrueckii* TdS6 and *S. cerevisiae* ScS13 added sequentially. SP: Spontaneous fermentation.

**Table 5:** Chemical parameters of wines produced in pilot scale fermentations (mean ± SD, *n* = 2 or *n* = 3 in IS and SMΤ). Values not sharing a letter are significantly different (*p* < 0.05).

Chemical Parameter	Inoculation Protocol ^1^			
	IS	SMT	SQT	SP
Total acidity (as tartaric acid g/L)	6.6 ± 0.1 ^ab^	6.7 ± 0.3 ^ab^	7.0 ± 0.2 ^a^	6.4 ± 0.2 ^b^
pH	3.14 ± 0.03 ^a^	3.11 ± 0.01 ^ab^	3.12 ± 0.00 ^ab^	3.00 ± 0.08 ^b^
Volatile acidity (as acetic acid g/L)	0.36 ± 0.05 ^b^	0.32 ± 0.02 ^b^	0.28 ± 0.00 ^b^	0.57 ± 0.02 ^a^
Total SO2 (mg/L)	99.6 ± 4.8 ^a^	103.7 ± 3.4 ^a^	87.3 ± 6.7 ^a^	96.6 ± 15.4 ^a^
Glucose (g/L)	<0.6	<0.6	<0.6	<0.6
Fructose (g/L)	<0.6	<0.6	0.9 ± 0.3 ^a^	0.7 ± 0.1 ^a^
Citric acid (mg/L)	504 ± 14 ^a^	503 ± 40 ^a^	504 ± 9 ^a^	493 ± 25 ^a^
Tartaric acid (g/L)	3.2 ± 0.3 ^a^	3.3 ± 0.2 ^a^	3.7 ± 0.4 ^a^	3.2 ± 0.5 ^a^
Malic acid (g/L)	1.7 ± 0.0 ^ab^	1.7 ± 0.1 ^a^	1.6 ± 0.1 ^b^	1.7 ± 0.0 ^ab^
Succinic acid (g/L)	0.7 ± 0.1 ^bc^	0.7 ± 0.1 ^ab^	0.9 ± 0.1 ^a^	0.6 ± 0.0 ^c^
Lactic acid (g/L)	<0.6	<0.6	<0.6	<0.6
Glycerol (g/L)	6.2 ± 0.2 ^b^	6.1 ± 0.5 ^ab^	6.6 ± 0.1 ^ab^	6.8 ± 0.0 ^a^
Acetic acid (mg/L)	273 ± 47 ^b^	208 ± 4 ^b^	188 ± 8 ^b^	509 ± 23 ^a^
Ethanol (g/L)	105.6 ± 0.8 ^a^	104.2 ± 8.0 ^a^	100.1 ± 4.5 ^a^	108.9 ± 1.6 ^a^

^1^ IS: *S. cerevisiae* ScS13; SMT: *T. delbrueckii* TdS6 and *S. cerevisiae* ScS13 added simultaneously; SQT: *T. delbrueckii* TdS6 and *S. cerevisiae* ScS13 added sequentially. SP: Spontaneous fermentation.

**Table 6:** Major volatiles (mg/L) of wines produced in in pilot scale fermentations (mean ± SD, *n* = 2 or *n* = 3 in IS and SMΤ). Values not sharing a letter are significantly different (*p* < 0.05).

Chemical Parameter	Inoculation Protocol ^1^			
	IS	SMT	SQT	SP
Acetaldehyde	14.0 ± 1.0 ^a^	12.5 ± 1.4 ^a^	15.0 ± 3.1 ^a^	16.0 ± 1.7 ^a^
Ethyl acetate	51.0 ± 1.2 ^a^	55.3 ± 3.8 ^a^	46.5 ± 7.3 ^a^	77.9 ± 4.3 ^b^
Methanol	39.6 ± 3.0 ^a^	43.5 ± 1.8 ^a^	42.9 ± 1.3 ^a^	45.1 ± 1.6 ^a^
Propanol	21.3 ± 1.5 ^b^	24.4 ± 1.4 ^ab^	26.1 ± 0.4 ^a^	22.2 ± 0.9 ^ab^
Isobutanol	13.9 ± 0.6 ^b^	17.5 ± 1.4 ^b^	23.6 ± 2.4 ^a^	17.3 ± 0.1 ^b^
2-Methyl-1-butanol	21.4 ± 0.9 ^b^	26.6 ± 1.8 ^a^	30.0 ± 0.1 ^a^	20.4 ± 1.2 ^b^
3-Methyl-1-butanol	144.0 ± 5.4 ^b^	169.8 ± 10.3 ^a^	169.2 ± 2.1 ^a^	129.2 ± 5.6 ^b^

^1^ IS: *S. cerevisiae* ScS13; SMT: *T. delbrueckii* TdS6 and *S. cerevisiae* ScS13 added simultaneously; SQT: *T. delbrueckii* TdS6 and *S. cerevisiae* ScS13 added sequentially. SP: Spontaneous fermentation.

**Table 7:** Minor volatiles (μg/L) ^1^ of wines produced in pilot scale fermentations (mean ± SD, *n* = 3 or *n* = 2 in SP). Values not sharing a letter are significantly different (*p* < 0.05).

Chemical Component	Inoculation Protocol ^2^			
	IS	SMT	SQT	SP
**Esters**				
Methyl acetate	16 ± 0 ^b^	16 ± 2 ^b^	10 ± 2 ^c^	23 ± 2 ^a^
Ethyl acetate	4417 ± 247 ^b^	4466 ± 154 ^b^	3919 ± 412 ^b^	6108 ± 399 ^a^
Propyl acetate	18 ± 2 ^ab^	20 ± 1 ^ab^	13 ± 0 ^b^	22 ± 3 ^a^
Isobutyl acetate	36 ± 3 ^b^	42 ± 5 ^ab^	28 ± 2 ^b^	52 ± 6 ^a^
Isoamyl acetate	3663 ± 422 ^a^	3922 ± 798 ^a^	2427 ± 348 ^a^	3940 ± 915 ^a^
Hexyl acetate	2386 ± 577 ^ab^	1889 ± 354 ^ab^	1484 ± 272 ^b^	3419 ± 312 ^a^
Phenylethyl acetate	936 ± 205 ^a^	1009 ± 104 ^a^	737 ± 154 ^a^	920 ± 33 ^a^
3-Hexen-1-ol acetate, (E)-	16 ± 2 ^ab^	16 ± 3 ^ab^	9 ± 0 ^b^	20 ± 1 ^a^
3-Hexen-1-ol acetate, (Z)-	16 ± 2 ^ab^	16 ± 3 ^ab^	11 ± 0 ^b^	20 ± 2 ^a^
Heptyl acetate	6 ± 1 ^a^	4 ± 0 ^a^	9 ± 2 a	8 ± 1 a
Ethyl propanoate	33 ± 2 ^b^	38 ± 3 ^b^	54 ± 8 ^a^	29 ± 2 ^b^
Ethyl isobutyrate	9 ± 2 ^bc^	12 ± 2 ^ab^	16 ± 1 ^a^	6 ± 0 ^c^
Ethyl butanoate	140 ± 12 ^a^	156 ± 19 ^a^	120 ± 13 ^a^	182 ± 21 ^a^
Ethyl 2-methylbutanoate	4 ± 1 ^ab^	4 ± 1 ^ab^	6 ± 0 ^a^	1 ± 0 ^b^
Ethyl isovalerate	7 ± 2 ^a^	7 ± 2 ^a^	8 ± 1 ^a^	3 ± 0 ^a^
Ethyl hexanoate	3153 ± 729 ^a^	2971 ± 609 ^a^	2765 ± 390 ^a^	4106 ± 635 ^a^
Ethyl heptanoate	4 ± 0 ^ab^	3 ± 1 ^b^	7 ± 1 ^a^	3 ± 0 ^b^
Ethyl lactate	34 ± 5 ^a^	40 ± 6 ^a^	49 ± 5 ^a^	49 ± 22 ^a^
Methyl octanoate	15 ± 6 ^a^	9 ± 3 ^a^	8 ± 2 ^a^	14 ± 4 ^a^
Ethyl octanoate	11,702 ± 5101 ^a^	6261 ± 2239 ^a^	5542 ± 2371 ^a^	9264 ± 3095 ^a^
Isoamyl hexanoate	23 ± 9 ^a^	14 ± 4 ^a^	13 ± 5 ^a^	15 ± 4 ^a^
Ethyl decanoate	6518 ± 2963 ^a^	2804 ± 2017 ^a^	1988 ± 1588 ^a^	2196 ± 1149 ^a^
3-Methylbutyl octanoate	115 ± 48 ^a^	60 ± 36 ^a^	25 ± 23 ^a^	41 ± 17 ^a^
Butanedioic acid, diethyl ester	10 ± 1 ^a^	11 ± 2 ^a^	13 ± 4 ^a^	6 ± 0 ^a^
Ethyl 9-decenoate	591 ± 268 ^a^	243 ± 152 ^a^	353 ± 275 ^a^	280 ± 63 ^a^
Ethyl dodecanoate	1063 ± 415 ^a^	705 ± 442 ^a^	206 ± 152 ^a^	528 ± 29 ^a^
Ester_1862 ^3^	91 ± 21 ^a^	71 ± 41 ^a^	30 ± 30 ^a^	40 ± 0 ^a^
Ethyl tetradecanoate	13 ± 3 ^a^	13 ± 3 ^a^	2 ± 3 ^b^	6 ± 0 ^ab^
Ethyl hexadecanoate	10 ± 4 ^a^	8 ± 2 ^a^	2 ± 3 ^a^	6 ± 2 ^a^
Ethyl 9-hexedecenoate	9 ± 2 ^a^	9 ± 2 ^a^	3 ± 4 ^a^	8 ± 2 ^a^
Ethyl furoate	7 ± 1 ^a^	7 ± 1 ^a^	8 ± 2 ^a^	10 ± 1 ^a^
**Alcohols**				
1-Propanol	24 ± 8 ^a^	26 ± 4 ^a^	28 ± 4 ^a^	33 ± 5 ^a^
2-Methyl-1-propanol	116 ± 25 ^a^	128 ± 18 ^a^	165 ± 5 ^a^	130 ± 21 ^a^
1-Butanol	6 ± 1 ^a^	7 ± 1 ^a^	7 ± 1 ^a^	8 ± 1 ^a^
Isoamyl alcohol	2468 ± 334 ^a^	2622 ± 160 ^a^	2703 ± 50 ^a^	2034 ± 191 ^a^
4-Methyl-1-pentanol	5 ± 0 ^a^	4 ± 1 ^a^	3 ± 0 ^a^	3 ± 0 ^a^
3-Methyl-1-pentanol	11 ± 1 ^a^	11 ± 1 ^a^	8 ± 0 ^a^	8 ± 2 ^a^
1-Hexanol	198 ± 6 ^b^	189 ± 5 ^b^	266 ± 29 ^a^	231 ± 15 ^ab^
3-Hexen-1-ol, (E)-	2 ± 0 ^a^	2 ± 0 ^a^	2 ± 0 ^a^	2 ± 0 ^a^
3-Hexen-1-ol, (Z)-	3 ± 0 ^a^	3 ± 0 ^a^	3 ± 2 ^a^	3 ± 0 ^a^
1-Heptanol	11 ± 2 ^a^	13 ± 1 ^a^	12 ± 1 ^a^	14 ± 6 ^a^
2-Ethyl-1-hexanol	5 ± 1 ^a^	7 ± 1 ^a^	5 ± 1 ^a^	2 ± 3 ^a^
2,3-Butanediol (isomer 1)	60 ± 36 ^a^	73 ± 6 ^a^	102 ± 7 ^a^	307 ± 246 ^a^
2,3-Butanediol (isomer 2)	16 ± 6 ^a^	19 ± 4 ^a^	26 ± 1 ^a^	74 ± 56 ^a^
1-Octanol	5 ± 0 ^a^	5 ± 1 ^a^	4 ± 1 ^a^	6 ± 1 ^a^
1-Nonanol	2 ± 2 ^a^	2 ± 2 ^a^	1 ± 1 ^a^	3 ± 0 ^a^
1-Decanol	3 ± 0 ^a^	3 ± 0 ^a^	3 ± 1 ^a^	1 ± 2 ^a^
Phenylethyl Alcohol	990 ± 187 ^a^	1072 ± 118 ^a^	1032 ± 91 ^a^	596 ± 35 ^b^
1-Dodecanol	10 ± 5 ^a^	7 ± 12 ^a^	5 ± 7 ^a^	0 ± 0 ^a^
**Acids**				
Acetic acid	94 ± 32 ^a^	87 ± 5 ^a^	47 ± 66 ^a^	353 ± 230 ^a^
Hexanoic acid	80 ± 5 ^b^	104 ± 24 ^a^	78 ± 9 ^b^	113 ± 0 ^a^
Octanoic acid	219 ± 38 ^a^	244 ± 22 ^a^	201 ± 22 ^a^	261 ± 6 ^a^
Decanoic acid	45 ± 8 ^a^	41 ± 36 ^a^	83 ± 4 ^a^	61 ± 4 ^a^
Carbonyl compounds				
Acetaldehyde	24 ± 2 ^a^	22 ± 2 ^a^	34 ± 5 ^a^	37 ± 9 ^a^
Benzaldehyde	6 ± 3 ^a^	4 ± 3 ^a^	4 ± 2 ^a^	1 ± 0 ^a^
2-Nonanone	11 ± 3 ^ab^	9 ± 1 ^b^	9 ± 1 ^b^	16 ± 0 ^a^
Decanal	0 ± 0 ^b^	5 ± 5 ^a^	5 ± 2 ^ab^	2 ± 3 ^ab^
4-Methylbenzaldehyde	2 ± 2 ^a^	1 ± 3 ^a^	2 ± 1 ^a^	0 ± 0 ^a^
**Terpenes**				
D-Limonene	4 ± 0 ^a^	6 ± 3 ^a^	7 ± 1 ^a^	2 ± 3 ^a^
Linalool	3 ± 0 ^a^	3 ± 0 ^a^	4 ± 1 ^a^	2 ± 0 ^a^
b-Citronellol	3 ± 0 ^a^	2 ± 0 ^a^	2 ± 1 ^a^	0 ± 0 ^b^
b-Damascenone	9 ± 2 ^a^	9 ± 1 ^a^	10 ± 0 ^a^	11 ± 1 ^a^
Geraniol	8 ± 2 ^a^	7 ± 3 ^a^	2 ± 1 ^a^	3 ± 0 ^a^
Nerolidol	5 ± 1 ^a^	6 ± 1 ^a^	13 ± 1 ^b^	5 ± 1 ^a^
**Other compounds**				
1,1-Diethoxy ethane	64 ± 10 ^a^	63 ± 5 ^a^	56 ± 6 ^a^	71 ± 2 ^a^

^1^ Concentrations relative to internal standard (3-pentanol). Expressed as the ratio of each compound peak area to that of internal standard multiplied by its concentration (1000 μg/L). ^2^ IS: *S. cerevisiae* ScS13; SMT: *T. delbrueckii* TdS6 and *S. cerevisiae* ScS13 added simultaneously; SQT: *T. delbrueckii* TdS6 and *S. cerevisiae* ScS13 added sequentially. SP: Spontaneous fermentation. ^3^ Unidentified ester. The number refers to the retention index.
